# Items

**Published:** 1868-11-15

**Authors:** 


					﻿J. Brown, M.B., now at Ashton, Lee Co., Ill., wishes us to
change the address of his Journal to that place, from his
former residence.
As we have fourteen J. Brown, M.D’s on our list, will he be
kind enough to say where he removed from ?
A. B. Shipman, M.B., of Syracuse, N. Y., died at Paris,
France, recently. Obituary notice next number.
				

